# Evading the AAV Immune Response in Mucopolysaccharidoses

**DOI:** 10.3390/ijms21103433

**Published:** 2020-05-13

**Authors:** Matthew Piechnik, Kazuki Sawamoto, Hidenori Ohnishi, Norio Kawamoto, Yasuhiko Ago, Shunji Tomatsu

**Affiliations:** 1 Nemours/Alfred I. duPont Hospital for Children, Wilmington, DE 19803, USA; piechnik@udel.edu (M.P.); kz.sawamoto@gmail.com (K.S.); 2 Department of Medical and Molecular Sciences, University of Delaware, Newark, DE 19716, USA; 3 Department of Pediatrics, Graduate School of Medicine, Gifu University, Gifu 501-1194, Japan; ohnishih@gifu-u.ac.jp (H.O.); noriok-gif@umin.ac.jp (N.K.); fromfutabadai@gmail.com (Y.A.); 4 Department of Pediatrics, Shimane University, Shimane 690-8504, Japan; 5 Department of Pediatrics, Thomas Jefferson University, Philadelphia, PA 19107, USA

**Keywords:** adeno-associated virus, mucopolysaccharidoses, immune response, antibody, immunosuppression

## Abstract

The humoral immune response elicited by adeno-associated virus (AAV)-mediated gene therapy for the treatment of mucopolysaccharidoses (MPS) poses a significant challenge to achieving therapeutic levels of transgene expression. Antibodies targeting the AAV capsid as well as the transgene product diminish the production of glycosaminoglycan (GAG)-degrading enzymes essential for the treatment of MPS. Patients who have antibodies against AAV capsid increase in number with age, serotype, and racial background and are excluded from the clinical trials at present. In addition, patients who have undergone AAV gene therapy are often excluded from the additional AAV gene therapy with the same serotype, since their acquired immune response (antibody) against AAV will limit further efficacy of treatment. Several methods are being developed to overcome this immune response, such as novel serotype design, antibody reduction by plasmapheresis and immunosuppression, and antibody evasion using empty capsids and enveloped AAV vectors. In this review, we examine the mechanisms of the anti-AAV humoral immune response and evaluate the strengths and weaknesses of current evasion strategies in order to provide an evidence-based recommendation on evading the immune response for future AAV-mediated gene therapies for MPS.

## 1. Introduction

Mucopolysaccharidoses (MPS) are a group of rare lysosomal storage disorders (LSDs) caused by a deficiency in an enzyme responsible for the catabolism of glycosaminoglycans (GAGs). Currently, there are seven types of MPS along with 11 subtypes defined by different deficient enzymes; each type of MPS corresponds to the accumulation of specific GAGs and unique clinical manifestations. However, organs typically affected include the brain, eyes, respiratory tract, heart, liver, spleen, bone, and cartilage [1,2]. Severe MPS phenotypes are associated with early death occurring within the first two decades of life [3,4,5,6,7,8], while others with an attenuated phenotype may have near-normal life expectancies [3,7,8,9]. Current therapy options for MPS patients include enzyme replacement therapy (ERT) and hematopoietic stem cell transplantation (HSCT) [2]. ERT was approved for MPS I, II, IVA, VI, and VII by the Food and Drug Administration; however, several limitations are observed: (1) weekly or biweekly infusions for 5–6 h, (2) high cost [10], (3) rapid clearance (a short half-life time, 35–60 min) [11], and (4) limited impact on CNS involvement and skeletal dysplasia [11,12]. HSCT for a patient with MPS I started in 1981 [13]. HSCT is considered the standard of care for patients with MPS IH and an optional treatment for those with Hurler/Scheie syndrome (MPS IH/S) and Scheie syndrome (MPS-IS) (attenuated phenotypes of MPS I), MPS II, MPS IVA, MPS VI, and MPS VII [14]. To date, more than 1000 patients with MPS have undergone HSCT to treat their disease [15]. However, HSCT has several critical issues: (1) finding the appropriate donor, (2) risks of graft versus host disease and rejection, (3) limited impact on bone lesions, and (4) the requirement of well-trained staff and facilities. Limitations of ERT and HSCT are well observed; therefore, novel therapeutic options with gene therapy are being pursued in preclinical and clinical trials (Table 1).

Gene therapy presents itself as a promising therapeutic option in producing functional enzymes in transduced cells. Several preclinical studies have documented success in delivering effective copies of the deficient gene into cells through recombinant viral vectors, including lentivirus (LV), retrovirus (RV), adenovirus, and adeno-associated virus (AAV) [27,28]. AAV is the most commonly used gene therapy vector because of its non-pathogenicity, long-term expression, and the availability of several different serotypes (each with different tissue tropism, immunogenicity, and efficiency) [27,29]. Currently, eight clinical trials with AAV vectors for MPS are underway in the United States [30].

AAV belongs to the *Dependovirus* genus in the Parvoviridae family and is a small, non-enveloped virus containing a single-stranded DNA genome. The wild-type AAV genome consists of two palindromic inverted terminal repeats (ITRs) flanking two open reading frames (ORFs), which code for the *rep* and *cap* genes, responsible for AAV genome replication and viral capsid protein production, respectively [31]. To be used as a gene therapy vector, the *rep* and *cap* genes are removed, allowing for a cassette to be used in place, with a maximum loading capacity of 4.7 kb [32,33]. The AAV vectors are then able to transduce human cells, with various AAV serotypes displaying optimal transduction of differing tissues. AAV8 has been shown to transduce liver cells 10- to 100-fold more efficiently than other serotypes [34,35], whereas AAV9 has demonstrated active crossing of the blood–brain barrier, targeting the central nervous system [36,37].

However, certain limitations of efficacy to AAV have been observed, with vector and transgene neutralization as a result of the humoral immune response being a significant barrier to effective treatment. Manno et al. reported that in an AAV2 hemophilia B canine model, neutralizing antibodies even at very low titers (1:10) significantly inhibit transduction [38]. Similarly, decreased transgene expression as a result of neutralizing antibodies was noted in AAV-treated MPS VI cats [39] and MPS I dogs [40]. The clinical significance of the immune response, however, is best observed in the recent CHAMPIONS clinical trial of SB-913, an AAV/zinc finger nuclease (ZFN)-mediated gene therapy for MPS II. Diminishing efficacy was reported in plasma iduronate-2-sulfate (IDS) activity with a correlating increase in the liver enzyme ALT, which may be due to a cytotoxic response against transduced liver cells [20]. In the related EMPOWERS study investigating AAV/ZFN vectors for MPS I treatment, leukocyte alpha-L-iduronidase (IDUA) activity was increased to normal levels; however, plasma activity and urine GAG assay revealed no significant change from baseline [17]. Other potential causes may be a low dosage of ZFN or low efficiency of gene editing with ZFN. Further analysis of optimal dosage with consideration for minimizing immunogenicity is necessary to optimize treatment results.

**Figure 1:**
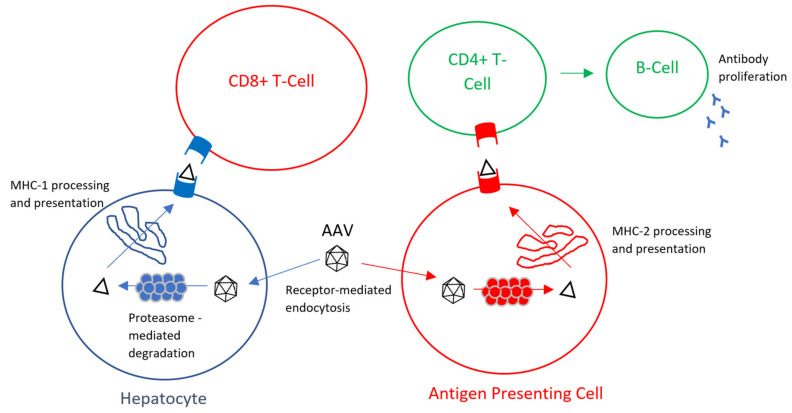
Mechanism of AAV CD8+ cytotoxic immune response and CD4+ humoral immune response [41].

In this review paper, we have reviewed the mechanisms of the AAV humoral immune response (Figure 1) as well as evaluated the current immune evasion strategies for MPS.

## 2. Anti-AAV Antibodies and the AAV Capsid

Since the 1960s, the humoral immune response to AAV has been studied, and anti-capsid antibodies raised were thought to be the primary cause of AAV transduction inefficiency [42]. Given the natural occurrence of AAV, children are often exposed to and generate antibodies against the AAV capsid early in their lifetime. Anti-AAV antibodies may also be prevalent at birth due to maternal transmission, and there is a progressive increase in anti-AAV antibody production through childhood into adolescence [43,44]. Geographic location is an essential factor for seroprevalence as well, with Calcedo et al. reporting that AAV seropositivity was observed ranging from 60% of African to 30% of American human serum samples [45]. Interestingly, there were no significant differences observed when comparing gender or race (Black, Caucasian, and Hispanic), as reported by Ellsworth et al. in their United States study [46]. In the MPS population, Fu et al. reported in a seroprevalence study comparing MPS types IIIA, IIB, and healthy children for seropositivity (≥1:50 titer) that no significant difference was apparent between the groups. Additionally, children younger than eight years old were observed to have significantly lower neutralizing AAV titers (serotypes 1–3, 5–9) compared to children older than eight years [47]. Therefore, the characteristics that primarily influence the seroprevalence of neutralizing AAV antibodies can be summarized as age and geographic location (Table 2).

The tendency for cross-reactivity against AAV compounds the problem of neutralizing antibody prevalence. Individuals often present with neutralizing antibodies against several different serotypes, likely due to homologies among the amino acid capsid sequence and the polyclonal response in humans [42,43,45,50,52]. AAV serotype clades are remarkably similar in their protein structure, with similarities ranging from 57–92% [53]. The relatedness of AAV capsid structures has led to several studies investigating homologies in the capsid surface and identifying distinct epitopes, which may be beneficial for immune evasion strategies. Different methods exist for identifying epitopes, including directed evolution, epitope searching, and biological structure-based approach [54]. A combination of these three strategies has resulted in the identification of numerous critical epitopes among the AAV serotypes that may prove useful in understanding their function and developing immune resistance. The Immune Epitope Database and Analysis Resource list: 11 epitopes for AAV1, 183 epitopes for AAV2, 42 epitopes for AAV4, 9 epitopes for AAV5, 2 epitopes for AAV7, 31 epitopes for AAV8 and 10 epitopes for AAV9 [55]. The epitopes identified include neutralizing as well as non-neutralizing antigenic sites, of which in recent years, the function has been studied. Non-neutralizing or binding antibodies attach to the AAV capsid at the sites that do not inhibit the normal function of the virus. Fitzpatrick et al. report that binding antibodies may enhance the tissue tropism and transduction efficiency of AAV vectors, an opposite effect of neutralizing antibodies [56]. Thus, the mechanism of capsid-targeting antibodies remains poorly defined, but avoiding the neutralizing effects of the humoral immune response provides an important consideration for AAV-mediated gene therapy.

## 3. Transgene Product Immune Response

The immune response against the transgene product plays a contributory role in reducing the global efficacy of AAV gene therapy. A reduction in circulating transgene product is observed, associated with an uptick in CD8^+^ T-cell concentration and the prevalence of anti-transgene product antibodies [52]. Although relatively limited in clinical trials due to restrictive exclusion criteria or immune-privileged tissue sites [57], transgene immune response and successive T-cell activation remain a prominent hurdle [57,58,59] to achieving maximal therapeutic efficacy.

In an MPS preclinical model, Hinderer et al. report in an MPS I canine model utilizing the AAV9 vector a substantial increase in anti-IDUA antibody titers in non-tolerized canines with a corresponding decrease in CSF IDUA activity [40]. There are limited data available in MPS clinical trials. In contrast, there is an extensive amount of work investigating hemophilia transgene product immunogenicity in clinical trials, which serves as a useful model for predicting immune response in MPS patients. In the first liver-targeted AAV gene therapy for hemophilia, Manno et al. demonstrated that over time the transgene product diminished as liver enzymes increased with a time course development consistent with a significant immune response [38]. This pattern of liver enzyme elevation was also observed in the MPS II clinical CHAMPIONS trial investigating in vivo ZFN packaged in AAV2/6 vectors. An increase in liver enzymes was noted after high levels of IDS were detected, which subsequently resulted in a decrease in enzyme activity [20]. Due to the delay in the liver enzyme elevation, it is possible that the immune response was generated against the transgene product and/or transduced cells as enzyme concentration increased. However, clinical trials have historically excluded patients who demonstrated adverse reactions or minimal efficacy to enzyme replacement therapy or similar treatments [20,38,52], which has limited the study of transgene product immunogenicity. It cannot be ruled out that the decrease in IDS activity could be due to low dosage or low frequency of gene editing. To determine the optimal dosage is critical as too low dose will not offer therapeutic benefit, whereas too high dose will elicit a strong immune response.

Despite the lack of full understanding, several advances in reducing the humoral response to transgene products have been made. The liver has demonstrated a vital ability to reduce the transgene product immune response when targeted by AAV gene therapy [60,61,62,63]. Several factors of viral vectors can affect its immunogenicity, with promoter selection being a prominent determinant. Pastore et al. demonstrated that the promoter selection in murine models could greatly influence humoral response with endogenous promoters to the liver, achieving a much lower rate of antibody production compared to ubiquitous promoters [64]. Exposure to antigens in the liver microenvironment tends to induce tolerance as opposed to eliciting an immune response [65]. The parenchymal, non-parenchymal, and lymphatic cells within the liver contribute to a complex relationship which results in T effector cell dysfunction and promotion of regulatory elements, allowing for adaptive immune tolerance [66]. Therefore, targeting the parenchymal liver cells ameliorates the deleterious humoral response. While the liver microenvironment remains not fully understood, recent advances in transcriptomic mapping utilizing single cell RNA sequencing have allowed for better characterization of the hepatic immune microenvironment [67]. These mappings may allow for further clarification on generating immune tolerance within the liver. Recently, Colella et al. designed a tandem promoter AAV therapy in a Pompe mouse model utilizing conjugated liver-muscle and liver-neuron promoters, which resulted in the successful prevention of a transgene product-directed immune response [60]. Liver-targeted AAV therapy provides the benefits for the treatment of MPS because of the liver capability to serve as an “enzyme factory.” The liver produces lysosomal enzymes at supraphysiological levels, which can be circulated and taken up by well-vascularized tissues allowing for “cross-correction” [27,68]. Therefore, utilization of the liver as a two-fold tolerance inducer [66] and foundational enzyme producer [68] may offer significant benefit to MPS patients, and therefore, clinical models should investigate this potential.

## 4. Evading the Anti-Capsid Immune Response

In evading the humoral response against the AAV capsid, several technologies have been developed to thwart the neutralization of AAV vectors. Three primary methods are discussed herein: (1) novel serotype development, (2) antibody reduction methods, and (3) antibody evasion tactics. 

Modifying the AAV capsid, specifically at epitopal sites, to reduce the neutralizing antibody targeting is achieved through strategies such as directed evolution [69], capsid chemical modification [70,71], and structure-based engineering [72,73]. There are over 100 current AAV serotypes [74], each with varying degrees of immunogenicity, tissue tropism, and efficacy as gene therapy. Directed evolution is a powerful tool for modifying the capsid to adapt against selective antibody pressure such as human sera. Tse et al. developed a novel AAV serotype that is not reliant on the selective pressures of neutralizing antibodies. Instead, they utilized CryoEM imaging accompanied by a directed evolution strategy to create a novel AAV1-derived serotype that effectively evades an anti-capsid immune response against human sera [73]. The availability of structural information is vital to the development of new capsids, and identification of epitopes and their homology will prove useful to further the generation of unique capsids.

Developing strategies to reduce the number of neutralizing antibodies within patients include the targeting of long-lived plasma cells and plasmapheresis. The production of antibodies by long-lived plasma cells (LLPCs) poses a significant challenge as LLPCs are privileged against immunosuppression and radiotherapy [75,76,77,78]. Velazquez et al. developed a mouse model to assess the benefits of using immunosuppressants (bortezomib, rapamycin, and prednisolone) individually and in combination with the clearance of neutralizing AAV9 antibodies. They determined that a combination of rapamycin and prednisolone could decrease the antibodies in serum by 85-93% after 8 weeks in addition to a significant decrease in B cells, plasma cells, and IgG and AAV9 specific antibody-secreting plasma cells [79]. Immunosuppression regimens are currently used in several clinical trials to mediate the immune response against AAV. In one clinical trial with the use of AAV8 vectors for delivery of hFIX gene to hepatocytes in hemophilia B patients, an elevation of ALT and AST liver enzymes was noted and was consistent with a capsid-specific CD8+ T cell activation against transduced liver cells [80,81]. The prompt use of oral corticosteroid ameliorated the immune response and preserved the transduced liver cells; however, the natural delay that occurs with recognition and proper treatment of the elevated liver enzymes was sufficient to cause significant reduction of transduced cells [58,80,81]. While effective, an increased risk of infectious disease for an already at-risk population may be detrimental to overall health, and more effective methods of immune response evasion are necessary. The potential for plasmapheresis as a method of reducing serum antibodies has also been investigated. Plasmapheresis is the process of removing blood from the patient and separating plasma and blood cells via centrifugation or filtration, then returning the blood cells and treated plasma or albumin saline solution to the patient. In a report by Monteilhet et al., they investigated the effect of plasmapheresis in 10 patients with <1:20 titer neutralizing factor against AAV types 1, 2, 6, and 8. With frequent plasmapheresis, multiple sessions with <5-day intervals between sessions, a significant reduction (between 1 and 64 fold for AAV1, 1 and 40 fold for AAV2, 2.5 and 20 fold for AAV6, and 1 and 20 fold for AAV8) was observed [82]. Therefore, the potential exists for plasmapheresis to lower neutralizing antibody (nAb) titers to levels that allow for inclusion in clinical trials. However, this immunosuppressive effect would likely be most significant in patients with inherently low titers yet still excluded from current trials. 

Antibody evasion tactics have been employed through methods such as exosome coated AAV vector, empty AAV capsid adsorption of antibodies, and varying drug delivery methods. Perhaps one of the most promising methods for antibody evasion is the development of AAV vectors coated by cell-derived extracellular vesicles. Exosomes, or microvesicles, are naturally occurring and membrane-derived vesicles that innately carry proteins and nucleic acids to neighboring cells [83]. As AAV vectors are non-enveloped virions that rely on capsid-surface interactions with antibodies to elicit an immune response, the production of an inert lipid-based envelope coating for the virus may shield them from the neutralizing effects of the immune system. Production of exosome-coated AAV vectors, as described by Maguire et al., involved transfecting 293T cells with a modified AAV2 pH22 (rep/cap expression vector) vector that digests the *cap* gene [84]. The 293T cells are then grown in media and for 48 hours, with a media exchange occurring at 16 hours for 2% exosome-depleted fetal bovine serum, after which the AAV vector-containing exosomes are isolated via gradient centrifugation [84]. Meliana et al. tested this theory by using liver-targeting coated and uncoated AAV vectors (AAV5 and AAV8) against hemophilia B liver cells with neutralizing AAV immune activity. Their findings showed that, against cohorts of varying titers (1:1, 1:1–1:3.16, and >1:10), both an amelioration of titer increase after subsequent antigen exposure and an increase in transgene product activity could be observed in cohorts with titers less than 1:3.16 [85]. While the ability for enveloped AAV vectors to largely evade the immune response is evident, higher titers of antibody completely neutralized the transgene product. In addition, the mechanism for transduction and immune evasion, as well as exo-AAV engineering, remains not well understood. Recently, improvements to exo-AAV production have been achieved [86] in addition to the development of fluoromicroscopy imaging techniques for exo-AAV activity, particularly within the brain [87]. Overall, the use of exosome coated AAV vectors may help to expand the number of patients eligible for gene therapy clinical trials. Other methods, such as administration of large doses of empty capsids, have attempted to “sponge” up the free-floating antibodies and allow for a greater percentage of drug-carrying vectors to target cells without neutralization [88]. The financial and practical challenge of mass-producing empty capsids, in addition to the risk of the unintentional ramping up of the immune response, may ultimately show that empty capsid dilution is a poor choice for evading the immune response.

## 5. Conclusions

The immune response to AAV vector-mediated gene therapy diminishes the efficacy of transgene production and limits the therapeutic success which we can achieve in current gene therapy. For MPS patients, AAV is a promising treatment for a challenging and difficult disease. By understanding the underlying physiology of the human immune response to viruses, we have been able to decipher the mechanisms involved in the AAV immune response. Critical elements such as capsid structure, transgene, and transduced cell behavior all play important roles in eliciting an immune response. Currently, a significant number of MPS patients have turned away from clinical trials due to pre-existing immunity to AAV serotypes. Still, in the early stages of AAV therapy, current clinical guidelines employ the use of immunosuppressants to overcome the immune response. However, as we progress in our understanding of AAV gene therapy, efforts to more efficiently overcome the immune response become necessary. Methods such as capsid engineering and chemical alteration, exosome-AAV production, novel immunosuppressant development, and plasmapheresis have been shown in preclinical and clinical models to achieve better immune response evasion. The utilization of these techniques may allow for more patients to enroll in clinical trials, furthering the advancement of AAV technology and the hopeful development of successful therapy. The current MPS clinical trials have shown promising early results. In the next wave of clinical models, the inclusion of immune response evasion tactics will become necessary to achieve the full potential of AAV gene therapy.

## Figures and Tables

**Table 1:** Current gene therapy clinical trials for mucopolysaccharidoses (MPS) [16,17,18,19,20,21,22,23,24,25,26].

MPS Type	Intervention	Company	Vector	Phase	Injection Method	Preliminary Data	Inclusion Criteria	Ref.
MPS I	RGX-111	REGENXBIO Inc.	AAV9	I/II	ICS	Expected 2nd half of 2020; inclusion criteria changed from >18 years to ≥4 months	CNS Involvement due to MPS I, 4 months and older, all sexes	[16]
	SB-318	Sangamo Therapeutics	AAV6/ZFN	I/II	IV	Increase in leukocyte IDUA activity into normal range. No change in plasma IDUA activity. No meaningful change in uGAG.	Clinical diagnosis of MPS I, ≥5 years, all sexes	[17]
	OTL-203	Orchard Therapeutics	Autologous HSC with lentiviral vector	I/II	IV	Expected 2nd half of 2020.	Biochemically and molecularly dx MPS IH, Lansky index >80%, indication to HSCT, lack of non-heterozygous IDUA HLA-matched sibling donor, 28 days to 11 years, all sexes	[18]
MPS II	RGX-121	REGENXBIO Inc.	AAV9	I/II	ICS	No SAEs reported. Mean reduction in CSF HS levels by 33.3% at Week 8. Stable neurocognitive development.	Documented diagnosis of MPS II AND neurocognitive testing score <77, 4 months to 5 years, male	[19]
	SB-913	Sangamo Therapeutics	AAV6/ZFN	I/II	IV	Small increases in IDS activity. Initial increase in plasma IDS activity, subsequent decrease due to transaminitis. No meaningful change in uGAG.	Male or female ≥5 years, clinical dx of MPS II base on clinical presentation, IDS deficiency confirmed by genetic sequencing	[20]
MPS IIIA	ABO-102	Abeona Therapeutics	AAV9	I/II	IV	Stable or improved neurocognitive development. Sustained reduction in CSF HS. No SAEs reported.	Dx of MPS IIIA by: no detectable or reduced SGSH, genomic DNA analysis w/ mutation in SGSH, 6mo to 2 years OR >2 years w/ DQ of ≥60	[21]
	OTL-201	Orchard Therapeutics	Autologous HSC with lentiviral vector	I/II	IV	None reported.	Normal cognition or mild deterioration of cognition, SGSH activity ≤10% of lower limit of normal, + normal activity of other sulfatase or mutation of SGSH, ≥3 months and ≤24 months, all sexes	[22]
	LYS-SAF-302	LYSOGENE	AAVrh10	II/III	IC	None reported.	Documented MPS IIIA diagnosis based on SGSH mutation genotyping, cognitive DQ score on BSID-III: 50% and above	[23]
	EGT-101	Esteve	AAV9	I/II	ICSF	None reported.	Under 18 years old, male and female, confirmed diagnosis of MPSIIIA	[24]
MPS IIIB	ABO-101	Abeona Therapeutics	AAV9	III	IV	None reported.	Confirmed dx of MPSIIIB by: no detectable NAGLU in plasma, genomic DNA analysis with homo/compound heterozygous mutations in NAGLU, 6 months to 2 years OR >2 years w/ cognitive DQ ≥60	[25]
MPS VI	AAV2/8.TBG.hARSB	FONDAZIONE TELETHON	AAV8	I/II	IV	None reported.	Documented biochemical and molecular dx of MPS VI, ≥4 year, Received ERT for 12 months prior, all sexes	[26]

IC: intracerebral; ICS: intracisternal; ICSF: intracerebro spinal fluid; IV: intravenous

**Table 2:** Seroprevalence studies among the human population in varying ages, geographic regions, diseases, and serotypes [47,48,49,50,51]. All studies are comprised of male and female subjects.

Geographic Region	Disease	Age	*n*	Titer Threshold	Anti-AAV Serotypes (%)									Ref.
					1	2	3	4	5	6	7	8	9	
China (Beijing, Anhui)	N/A	<18 years	37	1:10	-	100	-	-	40.5	-	-	67.6	-	[48]
	N/A	19–30 years	185	1:10	-	95.1	-	-	43.8	-	-	83.2	-	
	N/A	31–40 years	162	1:10	-	96.3	-	-	37	-	-	80.9	-	
	N/A	41–56 years	116	1:10	-	98.3	-	-	38.8	-	-	86.2	-	
United Kingdom	N/A	<6 months	129	1:5	-	-	-	-	-	-	-	10	-	[49]
	N/A	7 m–2 years		1:5	-	-	-	-	-	-	-	12	-	
	N/A	3–17 years		1:5	-	-	-	-	-	-	-	5	-	
	N/A	>18 years		1:5	-	-	-	-	-	-	-	43	-	
France	N/A	25–64 years	226	Unk.	67	72	-	-	40	46	-	38	47	[50]
United States	MPS IIIA	2–7 years	16	1:50	31	44	31	25	13	13	19	25	19	[47]
	MPS IIIA	>8 years	8	1:50	13	13	25	13	13	13	13	38	50	
	MPS IIIB	2–7 years	5	1:50	40	20	40	20	20	20	20	20	20	
	MPS IIIB	>8 years	9	1:50	0	11	33	11	0	11	11	11	0	
	N/A	2–7 years	18	1:50	6	17	22	22	6	17	11	17	6	
	N/A	>8 years	17	1:50	18	47	53	24	29	53	59	47	59	
Japan	N/A	>18 years	85	Unk.	36.5	35.3	-	-	37.6	-	-	32.9	36.5	[51]

## Abbreviations

AAV	adeno-associated virus
MPS	mucopolysaccharidoses
GAG	glycosaminoglycan
LSD	lysosomal storage sisorder
ERT	enzyme replacement therapy
HSCT	hematopoietic stem cell transplantation
IC	intracerebral
ICS	intracisternal
ICSF	intracerebrospinal fluid
IV	intravenous
CNS	central nervous system
IDUA	iduronidase
uGAG	urinary glycosaminoglycans
HS	heparan sulfate
CSF	cerebrospinal fluid
IDS	iduronate-2-sulfate
SGSH	N-sulfoglucosamine sulfohydrolase
DQ	developmental quotient
BSID	Bayley scale of infant development
NAGLU	N-acetyl-alpha-glucosaminidase
LV	lentivirus
RV	retrovirus
ITR	inverted terminal repeat
ORF	open reading frame
ZFN	zinc finger nuclease
Cryo-EM	cryogenic electron microscopy
ALT	alanine aminotransferase
AST	aspartate aminotransferase
MHC	major histocompatibility complex
LLPC	long-lived plasma cell
nAb	neutralizing antibody
